# SATB1 Knockdown Inhibits Proliferation and Invasion and Decreases Chemoradiation Resistance in Nasopharyngeal Carcinoma Cells by Reversing EMT and Suppressing MMP-9

**DOI:** 10.7150/ijms.49792

**Published:** 2021-01-01

**Authors:** Dongni Zhou, Chunsheng Ye, Zhiyong Pan, Yanfei Deng

**Affiliations:** 1 Department of Pathology, Zhongshan Hospital, Xiamen University, Xiamen, Fujian, China.; 2 Department of Otolaryngology-Head and Neck Surgery, Zhongshan Hospital, Xiamen University, Xiamen, Fujian, China.; 3 Union School of Clinical Medicine, Fujian Medical University, Fuzhou, Fujian, China.

**Keywords:** Nasopharyngeal carcinoma, SATB1, MMP-9, Vimentin, E-cadherin, EMT, Chemoradiation resistance

## Abstract

**Background:** Special AT-rich sequence binding protein 1 (SATB1) is a chromatin organizer and transcriptional regulator which regulate numerous cellular processes through effects on multiple gene expression. SATB1 is associated with drug resistance in several cancers. Whether SATB1 involves radiation resistance in nasopharyngeal carcinoma (NPC) and underlying mechanism of SATB1 to participate in chemoradiotherapy resistance in NPC have not been elaborated.

**Methods:** Chemoradioresistant NPC cell lines 5-8F/DDP (cisplatin) and 5-8F/R (radiation) were developed from 5-8F cell line. The expressions of SATB1, MMP-9 and EMT markers (Vimentin and E-cadherin) in these cell lines were examined by reverse transcription-quantitative (RT-q) PCR and western blot (WB) analysis. Cell viabilities of 5-8F/DDP treated with various concentrations of DDP and 5-8F/R irradiated with various doses of X-ray at the indicated time were investigated by MTT test. SATB1 was silenced in 5-8F/DDP and 5-8F/R cells by short hairpin RNA, and then the expressions of SATB1, MMP-9, Vimentin and E-cadherin were evaluated by RT-qPCR and WB analysis; the abilities of cell proliferation and invasion were assessed using MTT and transwell assays, respectively. Drug and radiation resistance assays were performed after SATB1 knockdown and cell viability was detected by MTT method.

**Results:** SATB1, MMP-9 and Vimentin were markedly upregulated in 5-8F/DDP and 5-8F/R cells compared with 5-8F cell, whereas E-cadherin was obviously downregulated. 5-8F/DDP and 5-8F/R cells displayed drug and radiation resistance to DDP or X-irradiation, respectively, while DDP or X-irradiation inhibited 5-8F cell viability in a time- and dose-dependent manner. Subsequently, knockdown of SATB1 resulted in decreased MMP-9 and Vimentin expression and increased E-cadherin expression in 5-8F/DDP and 5-8F/R. Furthermore, silencing of SATB1 suppressed proliferative and invasive abilities of 5-8F/DDP and 5-8F/R cells. Additionally, SATB1 knockdown reduced drug resistance of 5-8F/DDP cell to DDP and decreased radiation resistance of 5-8F/R cell to X-ray.

**Conclusion:** These results suggest that high expression of SATB1 plays an important role in the malignant behavior of NPC and leads to X-radiation and drug resistance in NPC through promoting EMT process and enhancing MMP-9 expression. SATB1 may be a promising therapeutic target for aggressive and chemoradiation resistant NPC.

## Introduction

Nasopharyngeal carcinoma (NPC) is an epithelial malignancy with high prevalence in southern China, southeast Asia, north Africa and some Arctic regions and shows strongly aggressive and metastatic biological properties, leading to a relapse or poor prognosis 1, 2. Until now, radiotherapy combined with chemotherapy remains as the preferred treatment for NPC 2, 3. Although great improvement has been achieved in therapeutic technology, residual or recurrent tumors remain as the outstanding cause of NPC treatment failure 2-4, especially for advanced stage patients. Alternatively, chemoradiation resistance is often induced and is a major factor leading to the failure of chemoradiotherapy and bad prognosis in NPC patients 2, 5, 6. Therefore, even some patients with the same clinical stages have different treatment outcomes. With the development of gene-based precision medicines, it is an urgent need to explore potential therapeutic targets for NPC and identify valuable biomarkers to predict the chemoradiotherapy resistance of NPC. Nevertheless, the study in this aspect remains limited.

The special AT-rich sequence binding protein 1 (SATB1), a transcription factor and a global genome organizer, is involved in pathogenesis and progression of numerous human cancers 7, 8. Aberrant expression of SATB1 has been found to be associated with proliferation, invasion, metastasis and poor prognosis in various cancers 8, 9. SATB1 was also identified as an independent prognostic marker in many types of cancer 8, 10. Furthermore, previous study found that SATB1 contributed to multidrug resistance (MDR) phenotype in breast cancer cells 11. Another study has confirmed that SATB1 contributes to MDR by inhibiting accumulation of vincristine (VCR) in gastric cancer cells and protecting the cells from VCR-induced apoptosis 12. Subsequently, our recent studies have revealed that SATB1 is obviously upregulated in primary NPC tissues and NPC cell line 5-8F and linked to drug resistance in NPC cell line CNE-2 13, 14. However, to our knowledge, no data are available on the relationship between SATB1 and radiation resistance in NPC. Additionally, the underlying mechanism of SATB1 to participate in chemoradiotherapy resistance in NPC is yet to be elucidated.

The epithelial-to-mesenchymal transition (EMT) is characterized as a transition from the epithelial cell phenotype into a mesenchymal phenotype. EMT is a crucial event leading to the initiation of invasion and metastasis for tumor progression and involved in chemoradioresistance of tumor cells, including NPC 15, 16. The characteristics of EMT are the loss of epithelial surface markers, most notably E-cadherin, and the acquisition of mesenchymal markers including Vimentin. E-cadherin and Vimentin were frequently dysregulated in multiple human cancers 16, 17. Recent findings have indicated that dysregulated SATB1 may mediate the reversion of EMT and mesenchymal-to-epithelial transition (MET) process and regulate its downstream target genes such as matrix metalloproteinases (MMPs; e.g. MMP-2, MMP-9) and EMT-related genes (e.g. E-cadherin, Vimentin) 18-20.

MMP-9, the important MMP family member, is a key protease to remodel and degradate extracellular matrix. It has been identified that MMP-9 plays a vital role in tumor development and progression 21. Further investigations have also implicated that MMP-9 is closely associated with chemoradiation resistance in certain tumors 22, 23 and participates in EMT process 24. For example, Asuthkar and his colleagues found that inhibition of MMP-9 resulted in suppression of EMT in medulloblastoma cells 25. However, to the best of our knowledge, no reports are available on the relationship between MMP-9 and chemoradiation resistance of NPC.

In this study, we developed radioresistant and chemoresistant NPC cell lines to detect the expression of SATB1, MMP-9, Vimentin and E-cadherin; and investigate the effect of SATB1 knockdown by short hairpin RNA (shRNA) on NPC cell proliferation, invasion, chemoradiation resistance; and identify the influence of SATB1 silencing on expression of MMP-9, E-cadherin and Vimentin; and then explore the possible mechanisms of above situations.

## Materials and Methods

### Cell lines and cell culture

The human NPC cell line 5-8F was cultured in RPMI-1640 medium (Gibco, Grand Island, NY, USA) supplemented with 10% fetal bovine serum (Invitrogen, Carlsbad, CA, USA), 100 IU/ml penicillin (Sigma-Aldrich Corp., St. Louis, MO, USA) and 100 μg/ml streptomycin (Sigma-Aldrich Corp.) at 37°C in a humidified atmosphere with 5% CO_2_. Exponentially growing cells were used for all experiments. 5-8F cell line used in this study was purchased from Cancer Institute, Central South University (Changsha, China).

### Establishment of a radioresistant cell line

The generated procedures of radioresistant subline of 5-8F were described and consulted in previous documents 26, 27. In brief, the cells were first grown to approximately 70% confluence in 25-cm^2^ culture flasks. Cells were initially irradiated with 2 Gy of X-ray from a linear accelerator (6 MV X-ray, UNIQUE™, Varian, Palo Alto, CA, USA) with a 1-cm tissue-equivalent bolus on top of the plate at a rate of 4 Gy/min and then returned to the incubator. These cells did not receive another exposure dose until 70% confluence was reached. The fractional dose was gradually increased each time until 10 Gy was reached. The total dose was 60 Gy. For the X-radiation treatment, cells were exposed to a desired dose, and were harvested in the indicated time points. The subline cells were named as 5-8F/R.

### Establishment of a chemoresistant cell line

The generated procedures of chemoresistant subline of 5-8F were described and consulted in previous documents 26, 27. The cisplatin (DDP)-resistant 5-8F subline was established by gradient induction. Briefly, 5-8F cells at log-growth phase were treated with 0.1 µg/ml DDP. Twenty-four hours later, DDP was removed and cells were washed by PBS. When cells' status returned to normal growth, they were passed for three generations followed by gradually increasing the concentration of DDP to 0.2, 0.5, 1.0 and 2.0 µg/ml until cells can maintain normal growth status. Those cells that can normally grow under the treatment of 2.0 µg/ml DDP were named as 5-8F/DDP.

### Cell viability assay prior to RNA interference (RNAi)

Each group (5-8F/DDP, 5-8F/R and the control 5-8/F) cells were seeded in 96-well plates at a density of 2 × 10^3^ per well, and cultured at 37°C in 5% CO_2_ for the indicated time (24, 48, 72 and 96 h)*.* At the different time points, cells were treated with DDP at different concentration (0.5, 1, 2 and 4 μg/ml) or X-irradiation at different dose (2, 4, 6 and 8 Gy), respectively. After treatment, the cells were incubated with 20 µl 5 mg/ml 3-(4,5-dimethylthiazol-2-yl)-2,5-diphenyltetrazolium bromide (MTT) solution reagent (Sigma-Aldrich Corp.) for 4 h, and then the cell viability was evaluated by MTT assay. The absorbance at 490 nm (A490) was measured using a microplate reader (Bio-Tek Inc., Winooski, VT, USA)*.*

### Reverse transcription-quantitative PCR (RT-qPCR)

Total RNA was extracted from cells using GeneJET™ RNA purification kit (Fermentas, Hanover, MD, USA) according to the manufacturer's instructions. With 1 µg of total RNA used as a template, cDNA was synthesized using RevertAid™ first strand cDNA synthesis kit (Fermentas). PCR was performed using the following conditions: pre-denaturation at 94°C for 2 min, followed by 35 cycles at 94°C for 30 s, 60°C for 30 s and 72°C for 30 s, with a final extension at 72°C for 5 min. Primer sequences used for RT-qPCR are listed in **Table 1**. The qPCR assays were performed using SYBR™ green PCR master mix (Applied Biosystems Inc., Foster City, CA, USA) on an ABI 7500 real-time PCR system (Applied Biosystems Inc.). GAPDH was used as the internal control. The relative mRNA expression levels were calculated using the 2^-ΔΔCt^ method.

### Western blot (WB)

Total protein was extracted using RIPA buffer (Sigma-Aldrich Corp.), and the protein concentrations were quantified by BCA protein assay kit (Boster Ltd., Wuhan, China). Total protein (50 μg) was separated by 10% SDS-PAGE and transferred onto polyvinylidene fluoride membranes (PVDF; Millipore, Billerica, MA, USA). The membranes were incubated with polyclonal rabbit anti-SATB1 antibody (1:2,000; Proteintech, Chicago, IL, USA), and then incubated with horseradish peroxidase-conjugated secondary antibody (1:5,000; Proteintech). The ECL-based detection was performed with enhanced chemiluminescent reagent kit (Pierce Biotechnology Inc., Rockford, IL, USA) according to the manufacturer's instructions. Protein bands were visualized using the ECL plus detection system (Pierce Biotechnology Inc.). The level of α-tubulin was used as a loading control.

### Transfection

SATB1 shRNA was designed and cloned into the pGFP-V-RS retroviral vector (Origene, Rockville, MD, USA). The SATB1-shRNA sequence was as follows: 5'-AGATTCAGCAGGAAATGAAGCGTGCTAAA-3'. The negative control shRNA sequence was as follows: 5'-AAGTCTTCTGACGCTGCTGCCTGGTCCAG-3'. Before transfection, 5-8F/DDP or 5-8F/R cells (5 × 10^5^/well) were seeded in 6-well plates and grown in culture medium without antibiotics. For transfection, 4 µg of the target plasmids and 10 µl of Lipofectamine™ 2000 (Invitrogen) were diluted separately with 250 µl of serum-free Opti-MEM™ (Gibco, Mulgrave, Victoria, Australia) medium, and then gently mixed. After 20 min, the mixture was added into the seeded cells at 80% confluence. After 5 h, the medium was replaced by normal medium. After 36 h incubation, the transfected cells were selected for 14 days with 0.5 µg/ml puromycin (Invitrogen). The cells with silenced SATB1 were designated as SATB1-shRNA group (SATB1-shRNA). Other groups were named as negative control group (NC-shRNA) and wild-type cell group (WT).

### Cell proliferation assay after RNAi

The cell proliferation ability was assessed by MTT assay. Each group (SATB1-shRNA, NC-shRNA and WT) cells were seeded in 96-well plates at a density of 2 × 10^3^ per well, and cultured at 37°C in 5% CO_2_ for the indicated time (24, 48, 72 and 96 h). At the different time points, cells were treated with 20µl MTT (5 mg/ml) solution reagent (Sigma-Aldrich Corp.). After incubation for 4 h, A490 was measured using a microplate reader (Bio-Tek Inc.).

### Cell invasion assay after RNAi

The cell invasion ability was determined using Matrigel Invasion Chamber (BD Biosciences, San Jose, CA, USA). In brief, cells in each group (SATB1-shRNA, NC-shRNA and WT) were inoculated in the upper chamber at a density of 5 × 10^4^ cells per 500 μl per chamber and maintained in serum-free medium, and lower chamber were filled with 750 μl complete medium. Cells were incubated for 24 h at 37°C in a 5% CO_2_ incubator. The invaded cells were fixed with 4% paraformaldehyde, and then stained with 0.1% crystal violet. Five random independent fields were counted under light microscope.

### Drug resistance assay after RNAi

After 5-8F/DDP cell was successfully transfected, each group (SATB1-shRNA, NC-shRNA and WT*)* cells were dispensed within 96-well plates at a density of 5 × 10^3^ per well and cultured at 37°C in 5% CO_2_*.* After 16 h, the cells were treated with 10 µl 1 μg/ml DDP. After 48 h incubation, cells were treated with 20 µl MTT (5 mg/ml) solution reagent (Sigma-Aldrich Corp.) for 4 h. The cell viability was assessed by MTT assay. A490 was measured using a microplate reader (Bio-Tek Inc.). The cell growth inhibition rate indirectly indicates the drug resistance ability of 5-8F/DDP cell to DDP.

### Radiation resistance assay after RNAi

After 5-8F/R cell was successfully transfected, each group (SATB1-shRNA, NC-shRNA and WT) cells were dispensed within 96-well plates at a density of 5 × 10^3^ per well and cultured at 37°C in 5% CO_2_ for 16 h*.* The cells were exposed to 4 Gy of X-ray for 48h and then cells were treated with 20 µl MTT (5 mg/ml) solution reagent (Sigma-Aldrich Corp.) for 4 h. Cell viability was measured by MTT assay. A490 was assayed using a microplate reader (Bio-Tek Inc.). The cell growth inhibition rate indirectly indicates the radiation resistance ability of 5-8F/R cell to X-irradiation.

### Statistical analysis

All measurement experiments were repeated three times independently, and the data are presented as mean ± standard deviation (SD). Comparisons between two groups were performed using Student's *t*-test. Comparisons among multiple groups were performed using ANOVA. Statistical significance was defined as a *P*-value<0.05.

## Results

### Chemoradiation resistance of 5-8F/DDP and 5-8F/R prior to SATB1 knockdown

**Figure 1:**
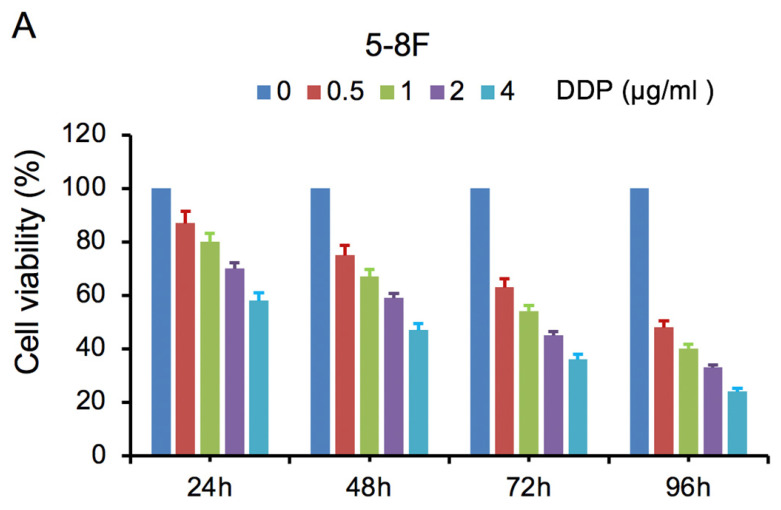

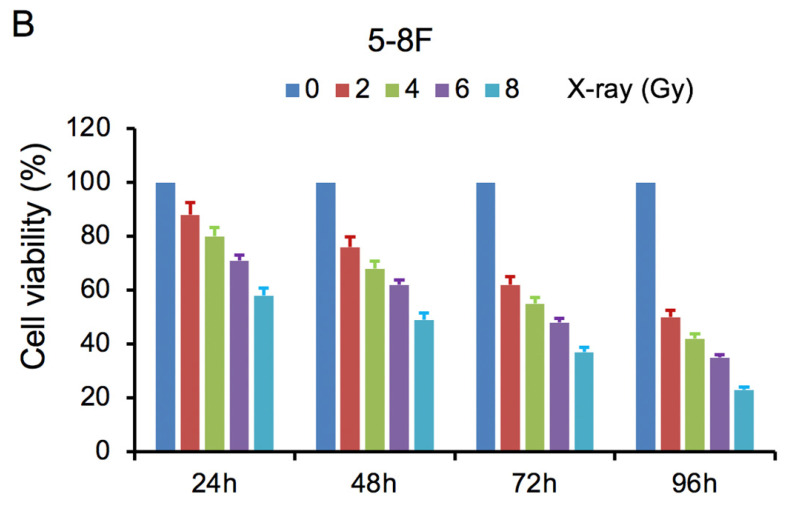

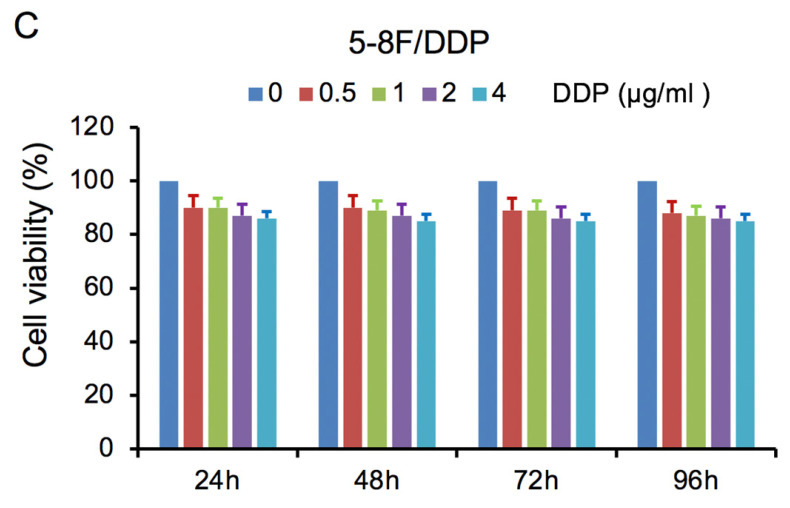

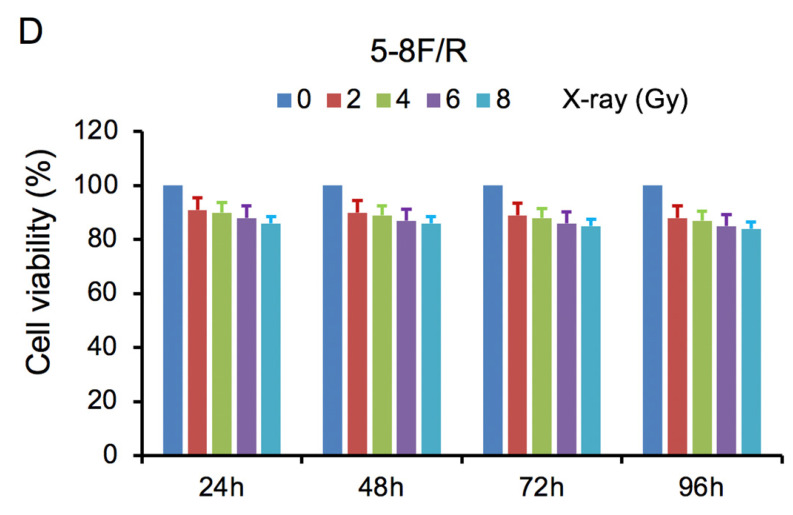
MTT cell viability assay showed that 5-8F/R and 5-8F/DDP were radioresistant or chemoresistant cells when compared to 5-8F cells. DDP (A) and X-irradiation (B) suppressed the cell viability of 5-8F in a dose- and time-dependent manner, respectively. 5-8F/DDP (C) or 5-8F/R (D) cells showed resistance to DDP or X-ray, respectively.

MTT cell viability assay was used to determine the chemoradiation resistance of 5-8F/DDP and 5-8F/R. The inhibitory effects of DDP on 5-8F and 5-8F/DDP cells were investigated by varying incubation times at drug concentrations of 0.5, 1, 2 and 4 μg/ml. The inhibitory effects of X-ray on 5-8F and 5-8F/R cells were investigated by varying incubation times at radiation doses of 2, 4, 6 and 8 Gy. As illustrated in **Figure 1A** and** 1B**, the 5-8F cells showed gradually reduced cell viability when treated with serial increased dose of DDP and X-ray or exposed to the indicated dose at serial increased times. Thus, DDP and X-irradiation caused an attenuation in the cell viability of 5-8F in a dose- and time-dependent manner, respectively. However, alteration of cell viability of 5-8F/DDP or 5-8F/R exposed to DDP or X-ray was not significant, respectively (*P*>0.05) (**Figure 1C**,** 1D**).

### Altered expression of SATB1, MMP-9, Vimentin and E-cadherin in chemoradiation resistant NPC cells

**Figure 2:**
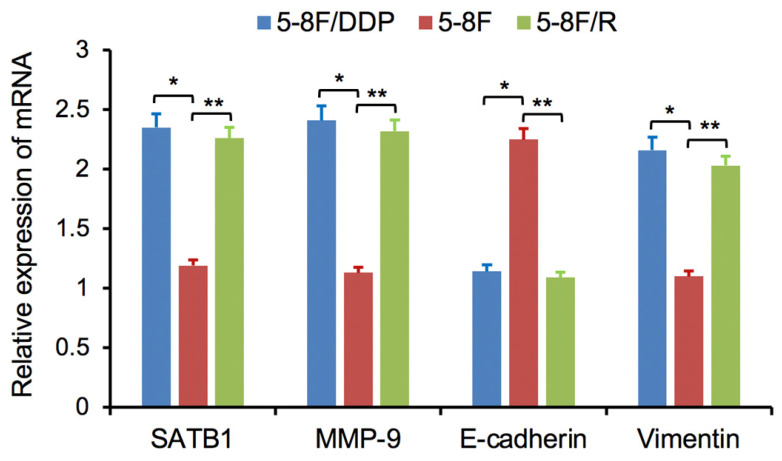
Expression of SATB1, MMP-9, Vimentin and E-cadherin genes in NPC cell lines by RT-qPCR. (* and **P<0.05 vs. 5-8F cell line).

**Figure 3:**
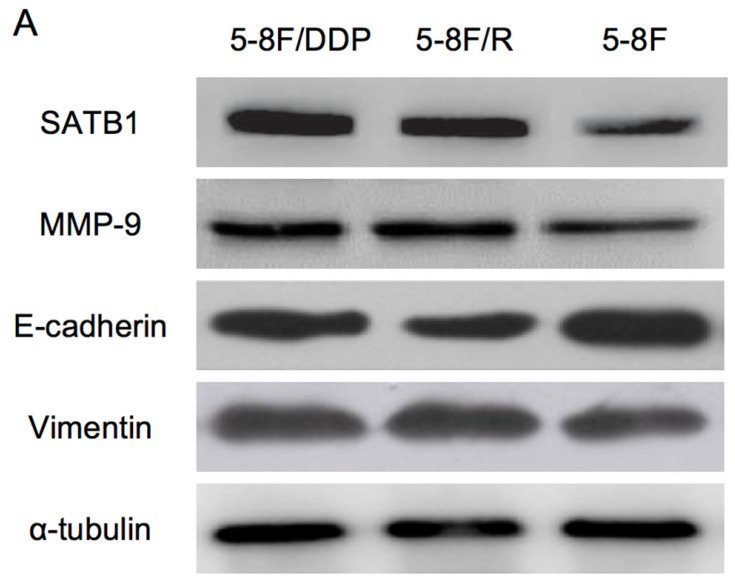

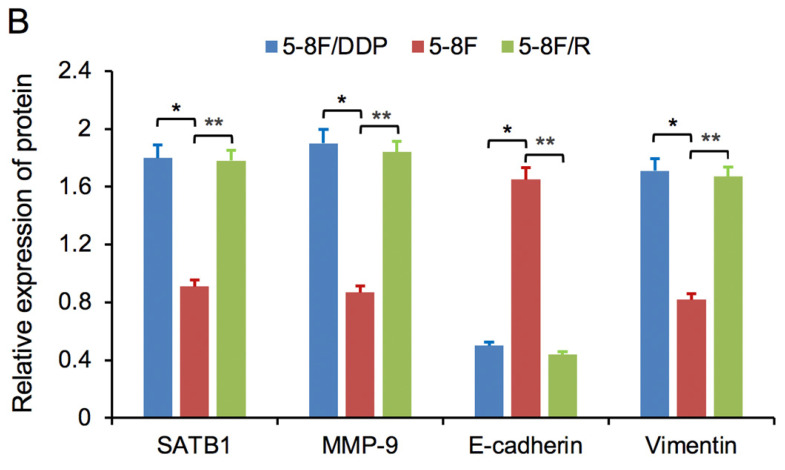
Expression of SATB1, MMP-9, Vimentin and E-cadherin in NPC cell lines by western blot analysis. (A) Representative protein electropherogram. (B) The relative protein expression levels of four genes normalized to that of α-tubulin. (* and **P<0.05 vs. 5-8F cell line).

RT-qPCR and WB analysis were used to assess the expression levels of SATB1, MMP-9, Vimentin and E-cadherin in 5-8F, 5-8F/DDP and 5-8F/R cells. RT-qPCR results showed that the mRNA expression levels of SATB1, MMP-9 and Vimentin in 5-8F/DDP and 5-8F/R were obviously higher than that in 5-8F (*P*<0.05) (**Figure 2**). Meanwhile, WB results showed that the protein expression levels of SATB1, MMP-9 and Vimentin in 5-8F/DDP and 5-8F/R were significantly increased than that in 5-8F (*P*<0.05) (**Figure 3A**,** 3B**). Nevertheless, the levels of mRNA and protein expression of E-cadherin in 5-8F/DDP and 5-8F/R were opposite compared with that in 5-8F (**Figure 2**,** 3A**,** 3B**).

### SATB1 was downregulated by RNAi in chemoradiation resistant NPC cells

**Figure 4:**
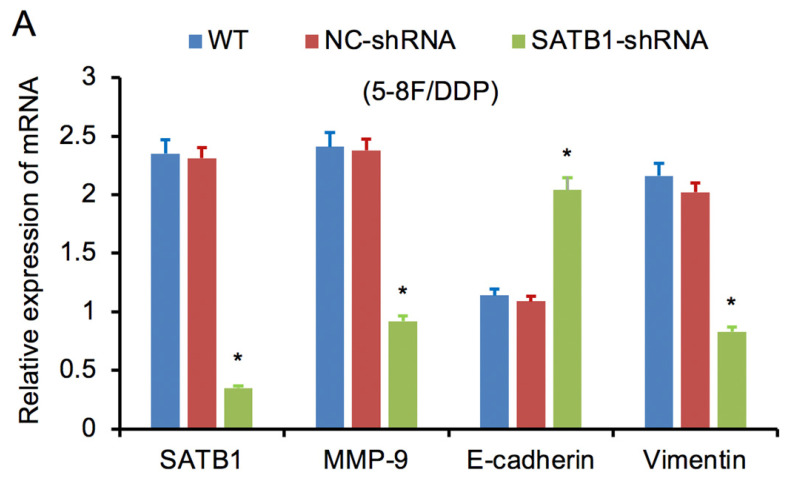

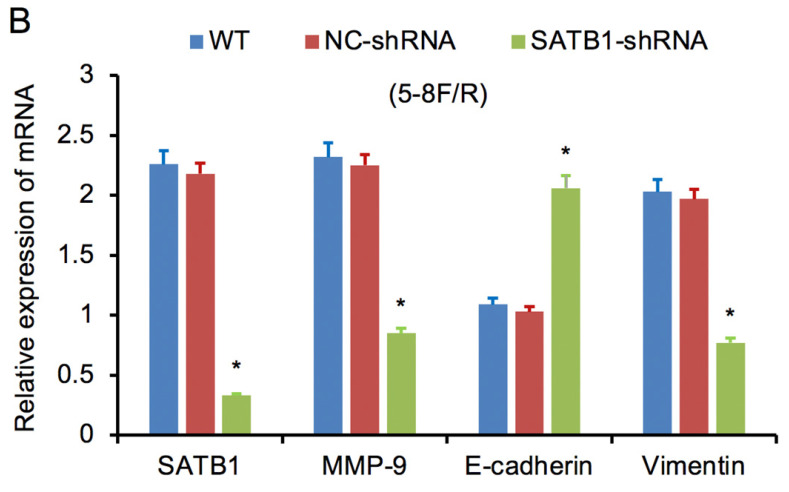
SATB1 knockdown regulated the expression of MMP-9, Vimentin and E-cadherin. (A) Relative mRNA expression levels of four genes in 5-8F/DDP cells. (B) Relative mRNA expression levels of four genes in 5-8F/R cells. WT, wild-type cell group (the untreated 5-8F/DDP or 5-8F/R cells). NC-shRNA, negative control group (the cells were transfected with negative control shRNA). (*P<0.05 vs. WT and NC-shRNA).

RT-qPCR and WB analysis were used to examine the silenced efficiency of SATB1 mRNA and protein expression in 5-8F/DDP and 5-8F/R cell lines. The relative expression levels of SATB1 mRNA and protein in SATB1-shRNA cells were evidently downregulated than those in WT and NC-shRNA cells (*P*<0.05) (**Figure 4A**,** 4B**, **5A-D**), while the difference of relative expression levels of SATB1 mRNA and protein between WT group and NC-shRNA group was not significant (*P*>0.05) (**Figure 4A**,** 4B**,** 5A-D**).

### SATB1 knockdown inhibited proliferation of chemoradiation resistant NPC cells

**Figure 6:**
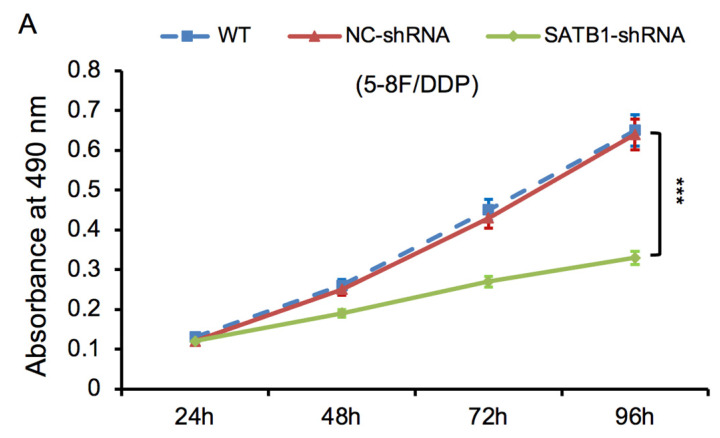

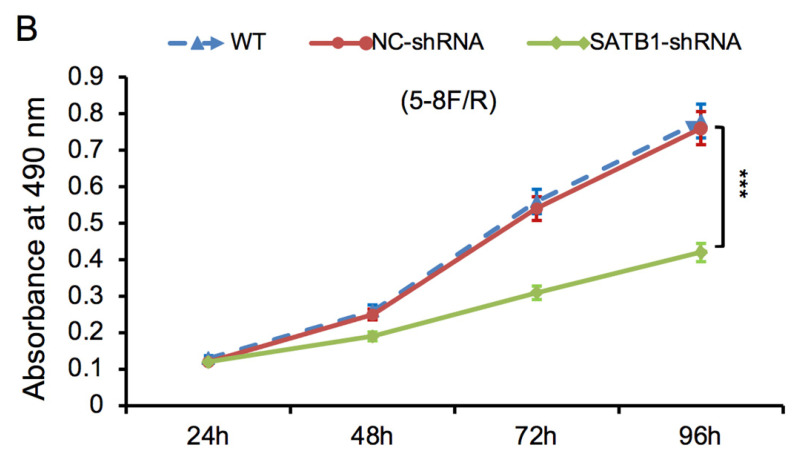
SATB1 knockdown inhibited proliferation in chemoradiation resistant NPC cell lines at different time-points (A) 5-8F/DDP cells and (B) 5-8F/R cells. Graph shows cell growth curve was drawn according to the A490 values at different time-points. (***P<0.05 vs. WT and NC-shRNA).

The proliferative ability of SATB1-shRNA, NC-shRNA and WT groups was examined by MTT method. The cell growth curve was drawn according to the A490 values at different time point, as shown in **Figure 6A** and **6B**. The proliferation rate of SATB1-shRNA group was lower than NC-shRNA and WT groups from 48 h, and the difference was significant (*P*<0.05).

### SATB1 knockdown inhibited invasion of chemoradiation resistant NPC cells

**Figure 7:**
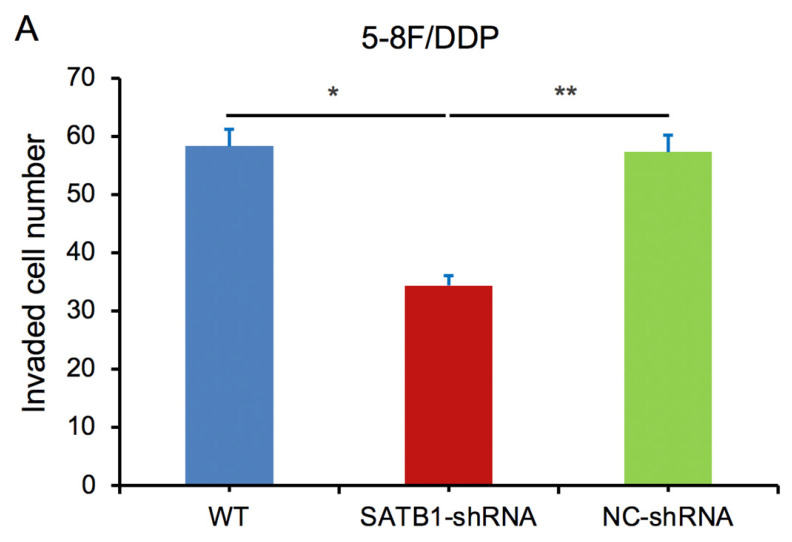

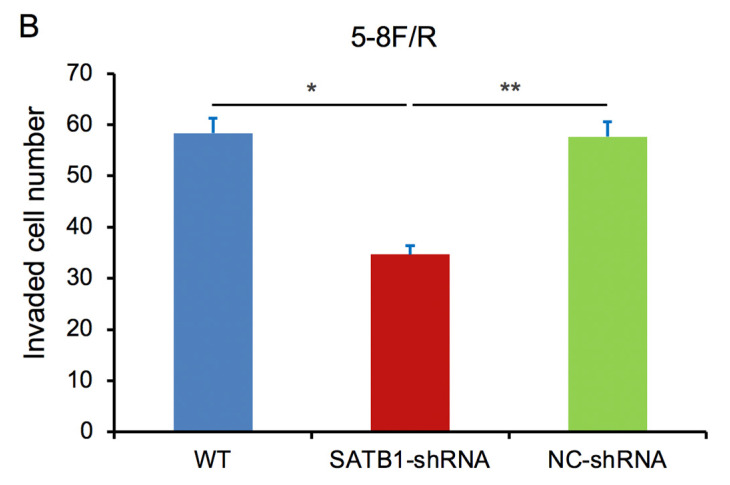
SATB1 knockdown inhibited invasion of chemoradiation resistant NPC cell lines (A) 5-8F/DDP cells and (B) 5-8F/R cells. Graph shows the number of invaded cells in the treatment groups for respective cell lines. (* and **P<0.05 vs. SATB1-shRNA).

In transwell assay, the transmembrane numbers of WT, SATB1-shRNA and NC-shRNA groups were shown in **Table 2**. Compared with NC-shRNA and WT groups, the transmembrane number of SATB1-shRNA group was obviously decreased (*P*<0.05) (**Figure 7A**,** 7B**).

### SATB1 knockdown modulated the expression of MMP-9 and EMT markers

RT-qPCR and WB analysis were used to assess the expression levels of SATB1, MMP-9, Vimentin and E-cadherin in 5-8F/DDP and 5-8F/R cell lines. The mRNA and protein expression levels of MMP-9 and Vimentin were downregulated along with knockdown of SATB1 (*P*<0.05) (**Figure 4A**,** 4B**,** 5C**,** 5D**). However, the mRNA and protein expression levels of E-cadherin were upregulated accompanied with knockdown of SATB1 (*P*<0.05) (**Figure 4A**,** 4B**,** 5C**, **5D**).

### SATB1 knockdown decreased 5-8F/DDP cell resistance to DDP and retarded 5-8F/R cell resistance to X-irradiation

**Figure 8:**
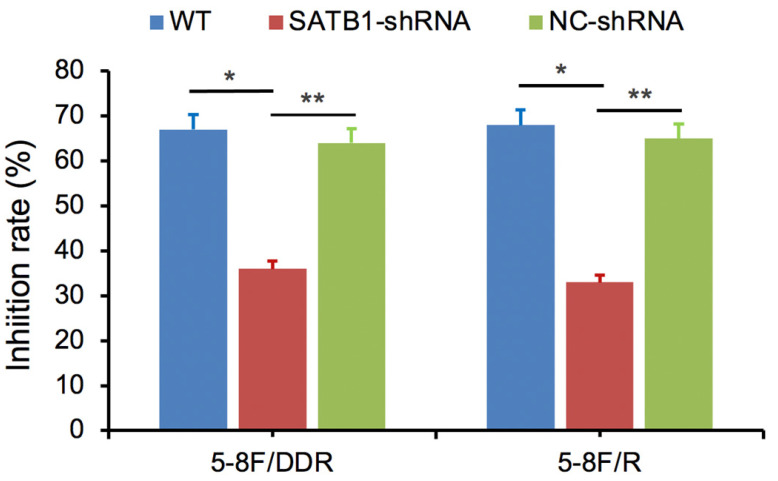
SATB1 knockdown decreased 5-8F/DDP cells resistance to DDP and retarded 5-8F/R cells resistance to X-irradiation. (* and **P<0.05 vs. SATB1-shRNA).

SATB1-silencing 5-8F/DDP or 5-8F/R cells (SATB1-shRNA), negative control cells (NC-shRNA) and WT cells were exposed individually to DDP (1 μg/ml) or X-irradiation (4 Gy). Cell viability was examined after 48 h and then the growth inhibition rate was calculated. Compared with NC-shRNA and WT groups, the inhibition rate of SATB1-shRNA group was significantly decreased (*P*<0.05) (**Figure 8**).

## Discussion

NPC is one of the common malignancies of epithelial origin in head and neck cancers 2, 28. Chemoradiotherapy is main treatment option for many epithelial tumor types including NPC, but the effectiveness of chemoradiotherapy is limited owing to radiation resistance and drug resistance 4, 6. Currently, there are few effective biomarkers available in the clinic for predicting tumor chemoradiosensitivity 1, 29, 30. To elucidate the underlying mechanism and find the novel therapeutic targets, the radioresistant and drug resistant cell lines were urgently needed. A DDP-resistant cell line 5-8F/DDP and a radioresistant cell line 5-8F/R have been established from human NPC cell line 5-8F. It is convenient for us to carry out subsequent experiments.

SATB1, a tissue-specific nuclear matrix-attachment DNA binding protein, is involved in chromatin structure packaging and gene expression and regulates numerous cellular processes such as differentiation, proliferation and apoptosis through effects on downstream gene expression 18, 31. SATB1 is an identified oncogene and its increased expression is associated with tumor growth, metastasis and poor prognosis in various cancers 7, 8, 32. Furthermore, it is an independent prognostic marker across several cancers 10, 33. Our previous study found that the expression levels of SATB1 were significantly upregulated in primary NPC tissues and NPC cell line 5-8F, and aberrant SATB1 expression was connected with Epstein-Barr virus (EBV) infection, metastasis and survival in NPC patients 13. Another investigation by our group indicated that SATB1 may be involved in the development, progression and drug resistance of NPC cell line CNE-2 14. Additionally, few works have demonstrated that STAB1 was closely associated with drug resistant and multidrug resistance in gastric cancer, breast cancer and human glioblastoma 11, 12, 34. To date, as we knowledge, no data on SATB1 and radioresistance of NPC were reported. In the present study, our results have displayed SATB1 expression levels of mRNA and protein were significantly elevated in NPC chemoradiation resistant cells 5-8F/R and 5-8F/DDP compared with 5-8F. This suggests that upregulated SATB1 may be induce the drug resistance and radiation resistance of NPC cells.

In next experiments, we successfully silenced the SATB1 expression in chemoradioresistant cell line 5-8F/DDP and 5-8F/R by shRNA. RT-qPCR and WB analysis showed the expression of SATB1 were distinctly downregulated after RNAi. MTT and transwell assays exhibited the capabilities of cell proliferation and invasion were visibly decreased after SABT1 knockdown. These results implicate that SATB1 promotes the growth and metastasis of NPC cells and successful silencing of SATB1 can prevent malignant behavior of NPC cells.

MMP-9 has been widely found to relate to the pathology of cancers, including but not limited to growth, invasion, metastasis and angiogenesis 21, 35, 36. Besides, previous works found that downregulation of MMP-9 can improve the chemosensitivity and radiosensitivity of some cancers 22, 23, 37. Next, recent studies indicated MMP-9 is downstream target molecule of some genes or signaling pathway, such as NF-κB, HIF, LINC00511 23, 38, 39. In this study, we found that MMP-9 was highly expressed in chemoradiation resistant NPC cells 5-8F/R and 5-8F/DDP compared with 5-8F, whereas its expression was remarkably decreased concomitant with silencing of SATB1. Above findings demonstrated that successful knockdown of STAB1 can suppress the expression of MMP-9 and MMP-9 may be the downstream target effector of STAB1.

Furthermore, MMP-9 has been shown to be involved in the progression of NPC via facilitating tumor growth, invasion and metastasis 37, 40, 41. Our previous study also found upregulated MMP-9 protein expression in NPC tissue was closely associated with EBV infection, metastasis, recurrence and poor survival of NPC patients 42. The present results first confirmed that abnormal MMP-9 expression contributes to cancer cell proliferation and invasion of chemoradiation resistant NPC.

In addition, various molecules including MMP-9 are known to regulate the EMT 24. Combined with our results regarding to MMP-9 and chemoradiation resistant NPC, it has been implicated that MMP-9 may be linked with NPC chemoresistance via participating in EMT processes 37, 43. Accumulating data have shown that EMT is closely associated with invasion, metastasis, radioresistance and chemoresistance in many cancers, including NPC 5, 17, 44. Previous study has documented that the development of DDP resistance in human NPC cells is accompanied by inducible EMT-like changes with an increased metastatic potential in vitro 43.

The reversible process of EMT is named as MET, which would allow transformed mesenchymal-like cells to regain their epithelial characteristics, a state that is perhaps more advantageous for survival in a foreign microenvironment 45. Reversion of expression of epithelial-related genes (e.g. E-cadherin) and mesenchymal-related genes (e.g. Vimentin) is the principal characteristic of EMT/MET transition. It has been reported that depletion of TAZ (Transcriptional co-activator with PDZ binding motif) reversed the EMT features to MET and restored the DDP sensitivity in resistant NPC cells 46. In the current study, high expression of Vimentin and low expression of E-cadherin in chemoradiation resistant NPC cells 5-8F/DDP and 5-8F/R were dramatically reversed along with knockdown of SATB1, and it was consistent with the alteration of MMP-9 expression. Thus, our results revealed that altered and reversed expression of MMP-9 and important EMT markers (E-cadherin and Vimentin) in chemoradiation resistant NPC cells, validating the roles of MMP-9 and EMT in SATB1-induced chemoradiation resistance of NPC. Taken together, these findings implied that depletion of SATB1 may reversed the EMT to MET situation by regulating its downstream genes controlling EMT processes.

Many studies have hinted the relationships among SATB1, MMP-9 and EMT. Han and his groups reported that SATB1 upregulated the expression of MMP-9 in breast cancer 18. A recent study found that SATB1 plays a crucial role in the progression of bladder cancer by regulating genes controlling EMT processes 20. SATB1 has also been identified to promote drug-induced EMT in breast cancer cell lines, driven by the positive feedback regulation of miR-448 and NF-κB signaling 47. Another study suggested that SATB1 may play an important role in the development and progression of liver cancer by main regulation of EMT 48. It's reported that SATB1 was able to promote the metastasis of prostate cancer by modulation of EMT 49. Moreover, Frömberg and colleagues revealed that knockdown of SATB1 in colorectal cell lines can simultaneously influence the expression of manifold genes, which involve EMT and matrix breakdown 50.

Therefore, we hypothesized that there may be a crucial regulatory relationship between SATB1 and MMP-9 and EMT during the formation of tumor growth, metastasis and chemoradioresistance in NPC. Our experiments have identified that SATB1, MMP-9 and Vimentin were upregulated in chemoradiation resistant NPC cells 5-8F/DDP and 5-8F/R compared with 5-8F, whereas E-cadherin was downregulated. Next works have confirmed that SATB1 knockdown in DDP-resistant and X-radiation resistant NPC cells caused downregulated expression of MMP-9 and Vimentin and increased expression of E-cadherin, and repressed cell proliferative and invasive ability. So, we deem SATB1 could induce chemoradioresistance of NPC cells and facilitate growth and metastasis of NPC through modulating MMP-9 expression and EMT process.

Additionally, the current results exhibited that DDP and X-ray individually did not prominently inhibit cell viability of 5-8F/DDP and 5-8F/R without SATB1 knockdown regardless of the increase of treatment dose and time. 5-8F/DDP or 5-8F/R cells showed resistance to DDP or X-ray, respectively. This is different from the effects of DDP and X-ray on non-chemoradiation resistant tumor cells 5-8F, which in a dose- and time-dependent manner. These data indicated drug or X-ray was difficult to improve therapeutic effect by increasing the dose of drug and X-irradiation when appearance of chemoradioresistance in NPC cells. It is helpful for clinical treatment decisions.

Subsequently, drug and radiation resistance assays demonstrated that cell viability of 5-8F/DDP and 5-8F/R with SATB1 knockdown was suppressed when 5-8F/DDP cell were treated with indicated DDP concentrations as well as the same situation in 5-8F/R cell treated with indicated X-ray dose. These results present that silencing of SATB1 can diminished drug resistance and radiation resistance in NPC cells.

In summary, SATB1 has been shown to contribute to tumor growth, metastasis, drug resistance and radiation resistance in NPC, and the possible mechanism is developed that SATB1 could simultaneously modulate multiple downstream factors, thus regulating tumor cell proliferation, invasion, chemoradiation resistance and EMT/MET switch. To the best of our knowledge, this is the first time that we here clarify the correlation between SATB1 and radiation resistance in NPC. Overall, our data indicate that SATB1 may be a promising therapeutic target and useful biomarker for chemoradiation resistant and aggressive NPC patients. Further studies are required to investigate clinical application value of SATB1 as the therapeutic target and predictive marker for NPC.

## Figures and Tables

**Figure 5:**
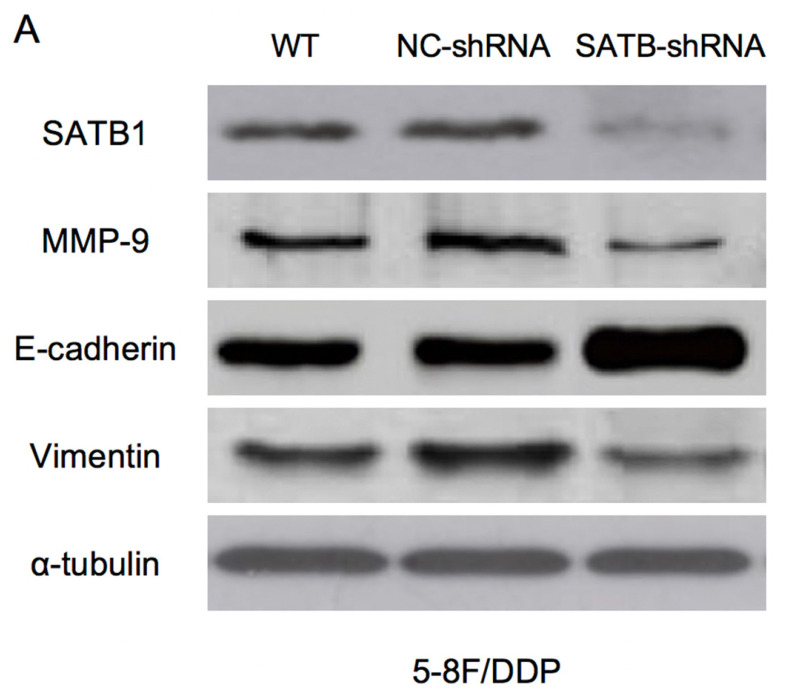

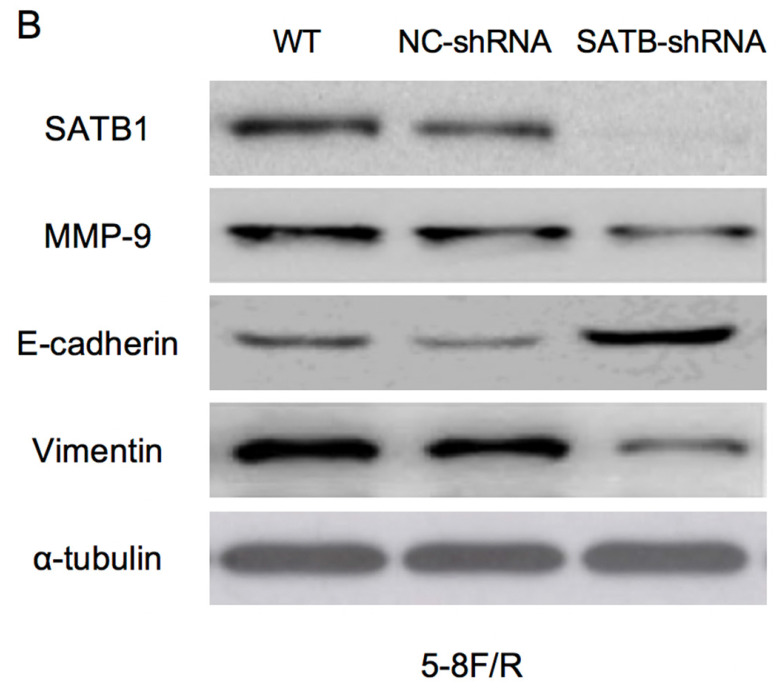

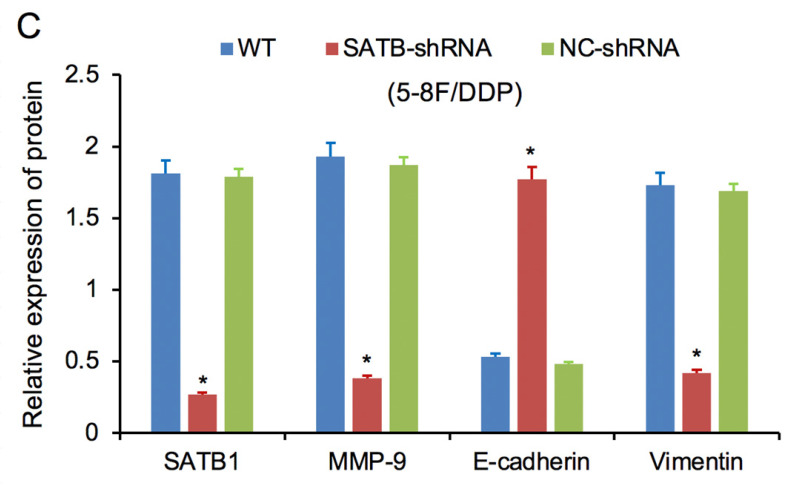

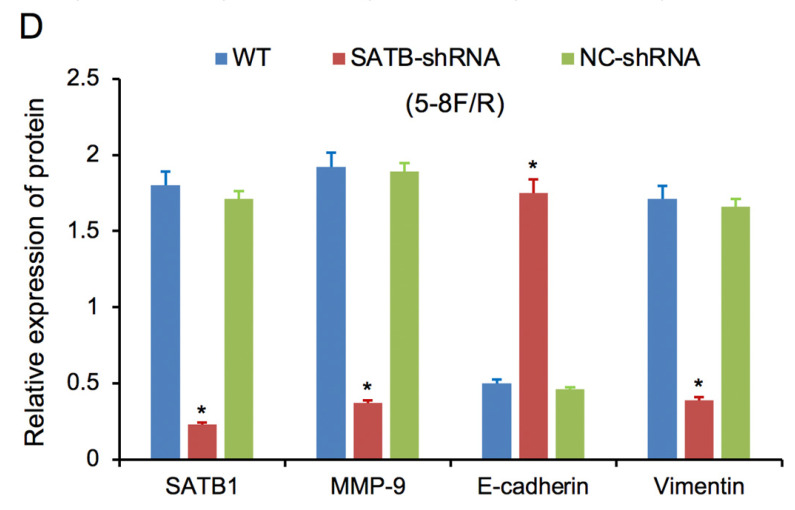
Expression of SATB1, MMP-9, Vimentin and E-cadherin in different groups by western blot analysis. (A) Representative protein electropherogram of 5-8F/DDP cells. (B) Representative protein electropherogram of 5-8F/R cells. (C) The relative protein expression levels of four genes normalized to that of α-tubulin in 5-8F/DDP cells. (D) The relative protein expression levels of four genes normalized to that of α-tubulin in 5-8F/R cells. (*P<0.05 vs. WT and NC-shRNA).

**Table 1:** Primer sequences used for RT-qPCR

Gene	Forward sequence (5'‑3')	Reverse sequence (5'‑3')
SATB1	GTGGAAGCCTTGGGAATCC	CTGACAGCTCTTCTTCTAGTT
MMP-9	GCATCCGAGCAAGAAGACAAC	CCCGACACACAGTAAGCATTC
E-cadherin	GAAGTGTCCGAGGACTTTGG	CAGTGTCTCTCCAAATCCGATA
Vimentin	AGATGGCCCTTGACATTGAG	TGGAAGAGGCAGAGAAATTC
GAPDH	TCGGAGTCAACGGATTTGGT	TTGGAGGGATCTCGCTCCT

**Table 2:** Quantification of invasion assay

Cell line	WT	SATB1-shRNA	NC-shRNA
5-8F/DDP	58.33±4.51	34.39±2.54	57.34±3.07
5-8F/R	58.38±4.55	34.66±2.61	57.65±3.12
